# In Situ Polymerization of Long Alkyl Chain Functional Groups Enhances the Oil–Water Separation Performance of Porous Organic Polymers

**DOI:** 10.3390/molecules30091925

**Published:** 2025-04-26

**Authors:** Hongbo Zhao, Shijie Cai, Ruoting Hua, Cong Li, Chunlong Xia, Bo Cui, Huimin Shao, Naishun Bu, Ye Yuan

**Affiliations:** 1 School of Environmental Science, Liaoning University, Shenyang 110036, China; zhaohongbozhb@126.com (H.Z.); c2950655949@163.com (S.C.); hrt010329@163.com (R.H.); 2 Fushun Hydrological Bureau of Liaoning Province, Fushun 113005, China; licong2008licong@163.com (C.L.); xiachunlong158@163.com (C.X.); 3 College of Chemistry, Liaoning University, Shenyang 110036, China; cuibo2019@163.com (B.C.);; 4 Key Laboratory of Polyoxometalate and Reticular Material Chemistry of Ministry of Education, Faculty of Chemistry, Northeast Normal University, Changchun 130024, China

**Keywords:** superhydrophobic surface, porous organic polymers, in situ polymerization, oil–water separation

## Abstract

The preparation of superhydrophobic functional materials is of great significance for applications in oil pollution control. However, the materials synthesized by traditional post-modification methods usually suffer from problems of limited active sites, uneven distribution, and susceptibility of the surface structure to external factors, which may significantly affect their superhydrophobic properties. In this study, the superhydrophobic porous organic polymer LNU-32 was successfully prepared via in situ polymerization with the introduction of green, low-surface-energy, long-alkyl-chain functional groups into the pores, which formed a “brush-like” structure on the pore surface of the polymer and effectively enhanced its hydrophobicity. The LNU-32 material exhibits excellent superhydrophobicity, with a water contact angle of more than 151°. In addition, the superhydrophobic polyester fabric prepared from LNU-32 has an oil–water separation efficiency of more than 90%. The adsorption capacity of the superhydrophobic fabric for dimethicone also reached 7.37 times its own weight. The study shows that the LNU-32 material exhibits good application potential in the field of oil–water separation, especially in the treatment of oily wastewater and oil spills.

## 1. Introduction

The discharge of petroleum pollutants and oily effluents into ecosystems can cause serious damage to biological habitats, not only inhibiting the growth and development of organisms and reducing soil fertility but also posing a potential threat to human health through bioaccumulation in the food chain [1]. At present, the main methods used to solve offshore oil spills include mechanical collection, in situ combustion, biodegradation, dispersant decomposition, sedimentation, and adsorption [2,3]. Among these, adsorptive separation has been widely adopted due to its cost-effectiveness, operational simplicity, and high remediation efficiency [4]. However, conventional adsorbents such as activated carbon and zeolites often suffer from drawbacks including high production costs and insufficient selectivity. Consequently, the development of adsorption materials capable of efficient oil–water separation is critical for treating oily wastewater. In recent years, porous organic polymers have shown good application prospects in the treatment of oil pollution due to their excellent adsorption performance, stable structure, and other characteristics. Porous organic polymers are a new type of polymeric materials characterized by a large specific surface area, an excellent porous structure, and abundant active and functional sites, and thus have great potential for application in the field of adsorption and separation [5,6]. Improving the hydrophobic properties of materials is usually achieved by introducing low-polarity functional groups, such as silane (–Si_n_H_2n+1_) or fluorine-containing groups, etc. [7,8,9,10]. Han et al. introduced fluorine-containing groups to prepare the hydrophobic material COF-DTF, and the COF-DTF@foam prepared from it exhibited an oil/organic solvent adsorption capacity of 69.7–173.2 times its own weight, showing good oil absorption performance [11]. Yu et al. prepared a superhydrophobic CMPs@SiO_2_ chromatographic stationary phase material, which demonstrated good hydrophobic properties and achieved the identification and separation of different oils [12]. However, silicon- and fluorine-containing groups have drawbacks such as non-renewability and biotoxicity [13]. In contrast, the long alkyl chain, as a key functional group of natural oils and bio-based substances, has good biocompatibility and degradability [14,15]. The incorporation of extended alkyl chains onto the pore surfaces of porous organic polymers could induce the formation of a brush-like polymeric architecture through molecular self-assembly processes. This structural reorganization subsequently facilitates the development of a conformal hydrophobic nonpolar coating along the pore walls, creating a continuous barrier layer through alkyl chain alignment. The existence of this thin layer significantly reduces the interaction force between the pores and water molecules and greatly increases the affinity for hydrophobic oil molecules [16,17]. Based on the above advantages, the long alkyl chain has the potential to replace the traditional low-surface-energy silicon- and fluorine-containing functional groups and can be used as a new type of functional group for the preparation of superhydrophobic interfaces.

Currently, the surface functionalization engineering of porous organic polymers is mainly based on two strategies, namely in situ polymerization and post-modification. The post-modification method usually grafts specific functional groups onto the pre-synthesized porous framework through chemical modification. However, this strategy is often limited by the steric hindrance effect of the modification sites, resulting in an uneven distribution of active sites and limited density of surface functional groups [18]. It is worth noting that solvent swelling and chemical treatment during the modification process can easily lead to the collapse of the framework structure, which significantly reduces the superhydrophobic properties of the materials. In contrast, the in situ polymerization strategy pre-introduces active units into the monomer structure through molecular design and realizes the precise positioning of functional groups during the polymerization process, constructing a topological network with predetermined functions in one step [19,20]. This synthetic route avoids complicated pretreatment and post-functionalization steps, and the process is highly integrated. It not only effectively maintains the integrity of the porous structure but also significantly improves the structural stability of the materials through the oriented arrangement of covalent bonds, while also showing good application potential [21].

In this study, based on the in situ polymerization strategy, the long alkyl chain functional groups with low surface energy were introduced into the pores via the Sonogashira–Hagihara coupling reaction, and the superhydrophobic porous organic polymer LNU-32 was successfully synthesized. The long alkyl chains build up a “brush-like” structure on the pore surface, forming a thin nonpolar layer, which significantly weakens the interaction force with water molecules and dramatically improves the hydrophobicity of the pore surface, and the water contact angle of the material is >151°. To solve the limitation of powder adsorbent application, this study used polyester fabric and polyurethane sponges as carriers to prepare superhydrophobic coating materials. The experimental results showed that the superhydrophobic coating material could adsorb dimethicone oil up to 7.37 g g^–1^, and the separation efficiency of all kinds of organic solvents exceeded 90%. By combining the intrinsic properties and hydrophobicity of porous organic polymers, this functional nano-coating is expected to have good application prospects in oil spill recovery and self-cleaning surfaces.

## 2. Results and Discussion

### 2.1. Structural Characterization of LNU-32

**Scheme 1:**
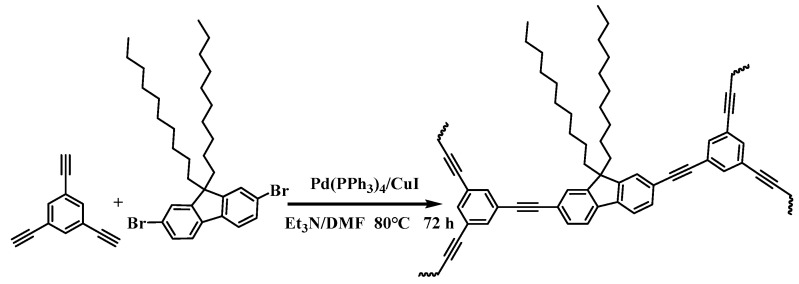
The synthesis of LNU-32.

**Figure 1:**
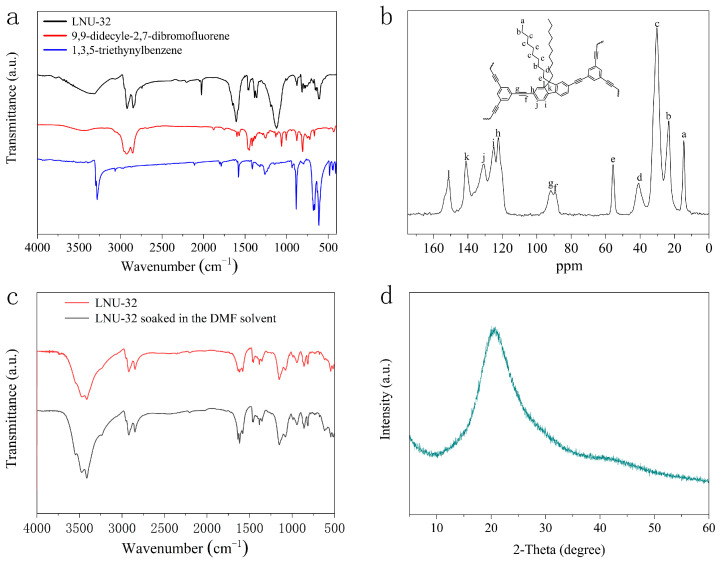
(**a**) FTIR spectra of LNU-32, 9,9-didecyle-2,7-dibromofluorene, and 1,3,5-triethynylbenzene. (**b**) ^13^C CP/MAS NMR spectrum of LNU-32. (**c**) FTIR spectra of LNU-32 and LNU-32 soaked in the DMF solvent. (**d**) PXRD pattern of LNU-32.

LNU-32 was synthesized by the Sonogashira–Hagihara coupling reaction of 2,7-dibromo-9,9-didecylfluorene and 1,3,5-triethynylbenzene. The original product was washed with chloroform, tetrahydrofuran, ethanol, and acetone, and further purified by Soxhlet extraction for four days using tetrahydrofuran, chloroform, and dichloromethane as solvents to produce LNU-32 (Scheme 1). The FT-IR spectrum of LNU-32 using the KBr pellet affirms the successful formation of the polymeric framework. In the FR-IR spectra, the disappearing characteristic signals at 500 (C–Br stretching band) and 3300 cm^–1^ (−C≡C−H stretching vibration) in the monomer and the appearing characteristic signals at 2200 cm^–1^ (–C≡C– stretching band) in LNU-32 (Figure 1a) demonstrated the completeness of the Sonogashira–Hagihara coupling reaction. Apart from that, a stretching band observed at ~1100 cm^–1^ was ascribed to the long alkyl chains compared with the monomer after the introduction of the crosslinking process. As illustrated in the ^13^C solid-state NMR spectrum, the signal peaks observed at 123–153 and 90 ppm were attributed to the resonances of aromatic carbons and –C≡C–, respectively. Additionally, five markedly broad peaks emerged at 23–57 ppm, which are assigned to the secondary carbon in the alkyl chain, while the peak observed at 14 ppm was attributed to primary carbon in the alkyl chain. This confirms the structural integrity of long alkyl chain fragments in the polymer (Figure 1b). All these results proved the successful occurrence of the Sonogashira–Hagihara reaction and the successful introduction of long alkyl chains. Furthermore, in order to ascertain whether the strong polar solvents would destroy the long alkyl chain structure, LNU-32 was immersed in N,N-dimethylformamide (DMF) for 24 h. Comparative Fourier transform infrared spectroscopy (FT-IR) analysis revealed that both pre- and post-treated LNU-32 samples exhibited characteristic stretching vibration absorption bands of long alkyl chains at approximately 1100 cm^–1^. This indicated that the long alkyl chains of LNU-32 revealed high stability in strongly polar environments (Figure 1c).

**Figure 2:**
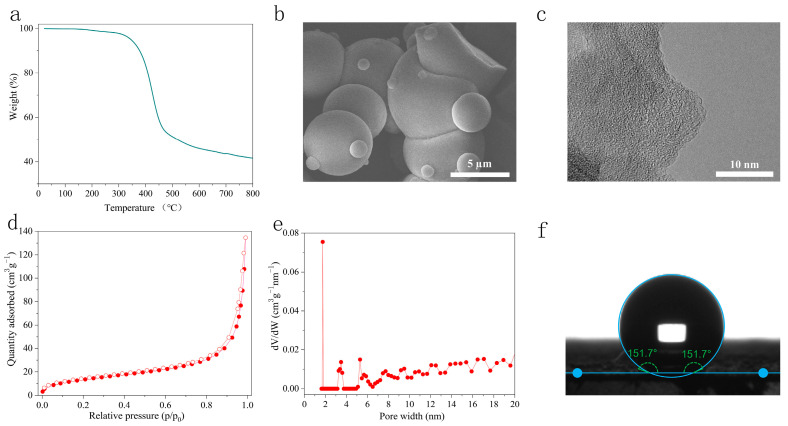
(**a**) TGA curve of LNU-32 under nitrogen conditions. (**b**) SEM image of LNU-32. (**c**) TEM image of LNU-32. (**d**) Nitrogen adsorption–desorption isotherms of LNU-32. (**e**) Pore size distribution of LNU-32. (**f**) WCA of LNU-32.

LNU-32 exhibits no indications of crystallization, which possessed an amorphous structure as revealed by the associated PXRD pattern (Figure 1d). This may be due to the fact that the dynamic twisting and winding properties of the long alkyl chains can interfere with the orderly stacking of the polymer backbone [22,23]. From the TGA curves, there is no obvious change for LNU-32 at 350 °C, exhibiting good thermal stability. The residual weight at 800 °C approached 38% for LNU-32 (Figure 2a). Alkyl chains decompose under the effect of heat [24], which may be the main reason for the significant weight loss of LNU-32 at high temperatures.

The scanning electron microscopy (SEM) images showed that the LNU-32 polymer exhibited a rough surface of irregular particles (Figure 2b). The stacking phenomenon of the particles leads to the loosening of the microstructure of LNU-32 and increases the additional surface area. In addition, the pile-up phenomenon increases the mesopores formed by the interstitial space, which greatly facilitates the absorption of oil molecules by LNU-32. Transmission electron microscopy (TEM) presented an ample worm-like pore channel structure inside LNU-32 (Figure 2c). This stacking morphology and pore structure allow the construction of surfaces with a high degree of roughness, and according to the lotus leaf effect, microscopically rough surfaces can enhance hydrophobic properties [25].

The porosity of the resulting polymer was investigated by N_2_ adsorption–desorption analysis at 77 K; LNU-32 belongs to the type II isotherm according to the IUPAC classification (Figure 2d). In the relative pressure range of 0.2–1.0, the adsorption and desorption of LNU-32 do not completely coincide, showing obvious hysteresis loops between them, which was attributed to the swelling effects in the presence of soft, porous organic materials [26]. In the high-pressure region of P/P_0_ > 0.8, the nitrogen adsorption isotherm has a steep upward trend, indicating the existence of mesopores and/or macropores in the network of LNU-32. Based on the non-local density functional theory (NL-DFT) model, the pore size of LNU-32 is mainly concentrated at 1.749 nm (Figure 2e). The calculated Brunauer–Emmett–Teller (BET) surface area of LNU-32 was 50.6208 m^2^/g. The small surface area of LNU-32 is attributed to the long alkyl chains occupying part of the pore space [27,28].

### 2.2. Superhydrophobicity of LNU-32

**Figure 3:**
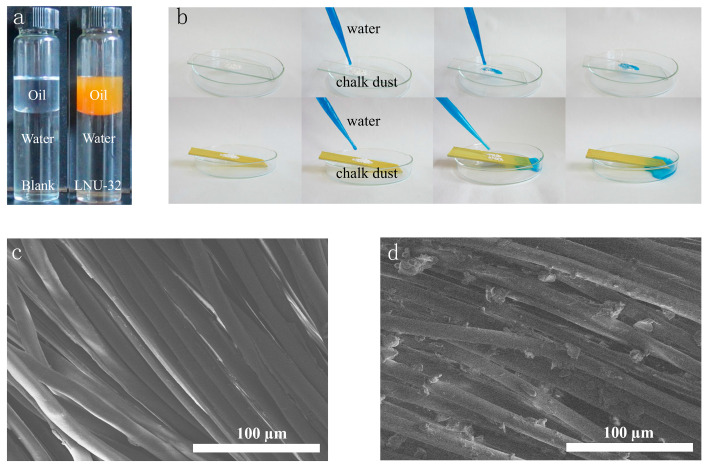
(**a**) Photograph of LNU-32 solids dispersed in a kerosene/water mixture. (**b**) Self-cleaning chalk dust performance of raw plate and LNU-32-coated plate. (**c**) SEM image of raw polyester fabric. (**d**) SEM image of LNU-32 located fabric.

The surface wettability was investigated by performing water contact angle measurements on the powder sample. LNU-32 exhibits a water contact angle (WCA) of 151.7° (Figure 2f), indicating its superhydrophobic character. Moreover, LNU-32 was dispersed in a mixture of kerosene and water (1:3 by volume). As shown in Figure 3a, the LNU-32 powder was found to be uniformly dispersed in the kerosene and remained above the water interface, further indicating the superhydrophobic and oleophilic character. The superhydrophobicity can be attributed to the long alkyl chains of LNU-32.

### 2.3. Self-Cleaning Performance of LNU-32

The self-cleaning properties of LNU-32 were investigated by applying LNU-32 powder to a glass plate. Chalkdust was used to simulate outdoor dust and sprinkled on the surface of the glass plate, which was then tilted at 20° to allow the water droplets to flow. The water droplets were stained with methyl blue for easy observation. Due to the strong affinity between the water droplets and the glass, the untreated glass plate adsorbed the chalk dust. For the LNU-32 coated glass plate, the chalk dust was carried away by the water droplets (Figure 3b). This phenomenon indicates that the glass plate coated with PAF solids has excellent superhydrophobicity and thus good self-cleaning ability.

### 2.4. Performance of LNU-32-Located Superhydrophobic Fabrics

Based on the above-mentioned investigations, we attempted to coat LNU-32 on a sponge and polyester fabric framework to realize oil/water separation through a simple operation and lower energy consumption process. The SEM images of the polyester fabric and LNU-32 coated polyester fabric show that the microstructure of the fabric was unchanged, but slight changes occurred on the fabric surface. The surface of the pristine fabric is smooth (Figure 3c,d), but the LNU-32-coated polyester fabric is uniformly covered with PAF particles [29].

**Figure 4:**
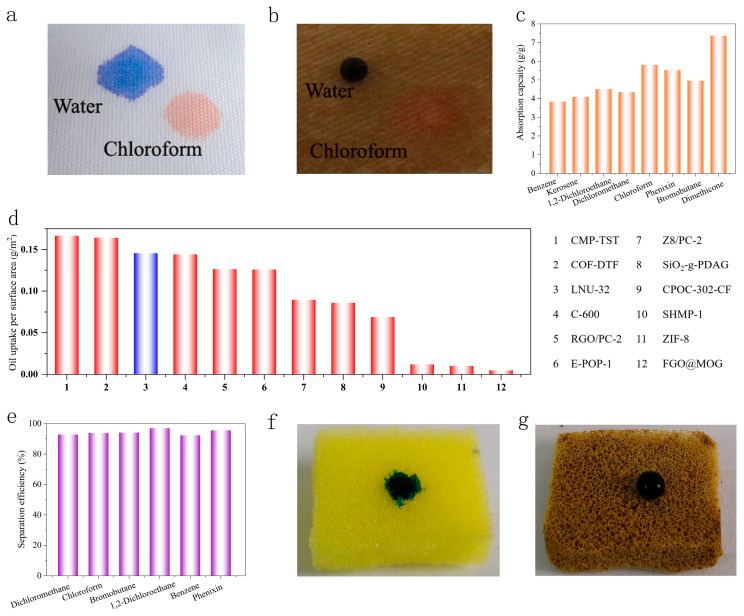
(**a**) Photograph of water and chloroform dripping on polyester fabric. (**b**) Photograph of water and chloroform dripping on LNU-32 located fabric. (**c**) Absorption capacity of LNU-32-loaded superhydrophobic polyester fabrics to different oils and organic solvents. (**d**) Oil uptake capacities of different adsorbents. (**e**) Separation efficiency of LNU-32 for different oil–water mixtures. (**f**) Photographs of water dropping on a polyurethane sponge and (**g**) LNU-32-coated sponge.

When water droplets (blue) and trichloromethane (red) were placed on the surface of a nonwoven fabric (white), they rapidly spread across the fabric surface and fully penetrated the material within 0.2 s. In contrast, when the same droplets came into contact with the LNU-32 coated polyester fabric, a distinct difference was observed: the water droplets maintained their spherical shape on the coated surface, while trichloromethane immediately permeated through the fabric (Figure 4a,b). This significant difference proves that after loading LNU-32, the wettability of the surface of the polyester fabric changes from hydrophilic to hydrophobic [30].

To further investigate the adsorption capacity of LNU-32 coated polyester fabric, the maximum adsorbing capacity of LNU-32 coated polyester fabric for various oils and organic solvents (Benzene, kerosene, 1,2-dichloroethane, dichloroethane, chloroform, phenixin, bromobutane, and dimethicone) was measured. Figure 4c presents the different adsorbing capacities of various oils and organic solvents. The difference stems from the interaction between the flexibility of the long alkyl chain of LNU-32 and the molecular structure of the adsorbates. The flexible alkyl chains entangle with organic molecules during adsorption, a process highly dependent on the adsorbate’s geometry. For instance, dimethicone’s extended, regular molecular chains align with the polymer’s alkyl chains, enabling smooth intercalation and effective entanglement, thereby maximizing adsorption. Conversely, benzene’s rigid planar ring structure hinders insertion into the polymer network, limiting interaction with the alkyl chains and resulting in reduced adsorption efficiency. By comparing this with existing studies, the maximum adsorption capacity per unit area of LNU-32 was slightly lower than that of CMP-TST(0.1665) and COF-DTF(0.164) but better than that of traditional materials such as C-600 (0.1443), SiO_2_-g-PDAG (0.0859), RGO/PC-2 (0.1262), E-POP-1 (0.1257), Z8/PC-2 (0.0895), CPOC-302-CF (0.069), SHMP-1 (0.0119), ZIF-8 (0.0102), FOG@MOG (0.0048), etc. (Figure 4d and Table 1) [31,32,33,34,35,36,37,38,39], demonstrating good adsorption ability.

### 2.5. Oil–Water Separation Properties of Superhydrophobic Polyester Fabrics

Dichloromethane, chloroform, bromobutane, 1,2-dichloroethane, benzene, and phenixin were used as test objects to study the separation efficiency of LNU-32. As shown in Figure 4e, the separation efficiency of LNU-32-coated polyester fabrics for several organic solvents is more than 90%. It is noteworthy that LNU-32 has the lowest separation efficiency for benzene, while it exhibits higher separation efficiency for oils with alkyl structures. Furthermore, as the length of the alkyl chain in the oil molecules increases, the separation efficiency of LNU-32 gradually improves. This phenomenon can be attributed to the entanglement effects between the alkyl chains [40], which enhance the interaction between LNU-32 and the alkyl-containing oil molecules.

### 2.6. Hydrophobic Properties of Modified Polyurethane Sponges

Sponges have attracted great attention in oil–water separation due to their low cost and high porosity, but the challenge of hydrophobic modification of sponges in a facile and scalable manner remains [41]. Like the method of preparing superhydrophobic polyester fabrics, the polyurethane sponge was modified by immersing the polyurethane sponge in a solution containing the superhydrophobic porous organic polymer LNU-32. When water was dropped on the surface of the pristine polyurethane sponge, it penetrated the sponge. In contrast, the water droplet presented a quasi-spherical shape on the modified sponge (Figure 4f,g). This proved that the modified polyurethane sponge has a strong repulsion effect on water and shows excellent hydrophobicity, and it is simple and feasible to modify the polyurethane sponge using this method, which shows broad application prospects.

## 3. Materials and Methods

### 3.1. Materials

1,3,5-Triethynylbenzene was provided by Tokyo Chemical Industry (Shanghai, China). 2,7-Dibromo-9,9-didecylfluorene was obtained from Energy Chemical Co. Ltd. (Shanghai, China). Cuprous iodide and tetrakis(triphenylphosphine)palladium were supplied by Sigma-Aldrich Corporation (Beijing, China). N,N′-Dimethylformamide and triethylamine were purchased from Sinopharm Chemical Reagent Co., Ltd. (Shanghai, China). All other materials were obtained from commercial suppliers and used without further purification.

### 3.2. Synthesis of LNU-32

LNU-32 was synthesized via the Sonogashira–Hagihara coupling reaction. 2,7-dibromo-9,9-didecylfluorene (96 mg, 4.50 mmol), 1,3,5-triethynylbenzene (150 mg, 0.99 mmol), cuprous iodide (10 mg), and tetrakis(triphenylphosphine)palladium (30 mg, 0.026 mmol) were dissolved in a mixture of triethylamine (8 mL) and N,N′-dimethylformamide (20 mL). The nitrogen degassing process was performed three times, and the mixture was heated to 80 °C for 72 h. After being cooled to room temperature, the mixture was washed with respective chloroform, tetrahydrofuran, ethanol, and acetone several times to remove the unreacted monomers and catalyst residues. Further purification was performed by Soxhlet extraction (tetrahydrofuran, chloroform, and dichloromethane) for 72 h, followed by drying at 90 °C for 24 h. The brown solid powder obtained was the target product, the porous organic polymer LNU-32.

### 3.3. Characterization

Fourier transform infrared (FT-IR) spectra were performed using KBr pellets on a Shimadzu-Prestige 21 Fourier transform infrared spectrometer (Shimadzu Corporation, Kyoto, Japan). Solid-state ^13^C CP/ MAS NMR spectra were measured on a Bruker AVANCE III 400 WB spectrometer at a MAS rate of 5 kHz (Bruker Switzerland AG, Zurich, Switzerland). Thermogravimetric analysis (TGA) was conducted using a METTLER TOLEDO TGA/DSC 2 thermal analyzer under an air atmosphere at a heating rate of 5 °C min^−1^ (METTLER TOLEDO, Zurich, Switzerland). Powder X-ray diffraction (PXRD) was performed by a Bruker D8 AVANCE diffractometer using Cu-Kαradiation (40 kV and 200 mA) with a 2θ range of 5~60° (Bruker Switzerland AG, Zurich, Switzerland). Scanning electron microscopy (SEM) analysis was performed on a SU8010 model scanning electron microscope with an accelerating voltage of 5 kV (Hitachi, Ltd., Tokyo, Japan). Transmission electron microscopy (TEM) was performed on a JEM-2100 with an accelerating voltage of 200 kV (JEOL Ltd., Tokyo, Japan). N_2_ adsorption isotherms were obtained on the American Mike TriStar-3020 gas sorption analyzer at 77 K (Mushroom Wisdom, Inc., Norcross, GA, USA). The contact angle of hydrophobicity was measured on the Krüss GmbH DSA1005 (KRÜSS GmbH, Hamburg, Germany).

### 3.4. Measurement of Water Contact Angle

The water contact angle (WCA) was measured on the Krüss GmbH DSA1005 (KRÜSS GmbH, Hamburg, Germany). The image was taken from 2 μL deionized water droplets on the polymer surface at room temperature within the first 5 s of dropping onto the polymer surface using a micro-syringe. It should be noted that the reported result is the average value of the repeated measurements from at least five different points on the sample to reduce the experimental error.

### 3.5. Preparation of the Superhydrophobic Polyester Fabric

A piece of polyester fabric (50 × 50 mm) was ultrasonically cleaned in ethanol, deionized water, and acetone (40 mL) for 30 min to remove any stains and oils. After that, the fabric was dried in an oven at 60–70 °C and then immersed in a 20 mL dichloromethane solution containing 20 mg of LNU-32. The fabric was sonicated in a dichloromethane solution for 1 h and dried to obtain a superhydrophobic polyester fabric coated with LNU-32.

### 3.6. Oil Spill Absorption

Adsorption tests were conducted using dimethyl silicone oil, kerosene, dichloromethane, 1,2-dichloroethane, bromobutane, chloroform, benzene, carbon tetrachloride, and chlorobenzene as representative organic solvents. The pre-weighed LNU-32-coated fabric (*M*_0_, mg) was immersed in oil–water mixtures until saturation (1 min), then reweighed (*M*_1_, mg). The adsorption capacity (*K*) was calculated as Equation (1):

(1) $$K=\frac{M_{0}-M_{1}}{M_{0}}$$

Separation efficiency test: The oil–water mixtures were prepared via homogeneous mixing of distilled water with dichloromethane, chloroform, bromobutane, 1,2-dichloroethane, benzene, and phenixin in equal volumes. The mixture of oil or organic solvent and water was subjected to ultrasonication until the two liquids were thoroughly amalgamated, and the fabric was first placed in the middle of the filtering apparatus and then the oil/water mixture was poured from the top. Gravity is the only driving force for separation during the separation process. The permeation flux of LNU-32-coated fabric was about 0.00025 m/s, and we calculated separation efficiency by determining the mass of organic solvent before and after separation. The oil/water separation efficiency (*η*, %) of the LNU-32-coated fabric was determined and calculated by Equation (2):

(2) $$\eta =\frac{m_{1}}{m_{0}}\times 100\%$$

In the formula, *m*_0_ (mg) represents the initial mass of the organic solvent and *m*_1_ (mg) represents the mass of the organic solvent collected from the mixed solution.

## 4. Conclusions

In this work, we used 2,7-dibromo-9,9-didecylfluorene building monomers to modify long alkyl chain fragments in situ in porous organic polymer cavities to construct superhydrophobic surfaces. The long alkyl chains aligned on the pore surface formed a thin non-polar layer, which significantly weakened the interaction force between the pore surface and water molecules and greatly improved the hydrophobicity of the pore surface with a water contact angle of >151°. By combining LNU-32 with substrates such as fabrics and sponges, superhydrophobic coating materials can be prepared by an impregnation process with excellent water resistance and self-cleaning properties. The modified polyester fabric showed good adsorption and separation ability, with an adsorption capacity of 7.37 g/g for dimethyl siloxane oil and a separation efficiency of more than 90% for various organic solvents. The present work provides a new way to prepare superhydrophobic surfaces, showing great potential for the large-scale removal of organic matter or oil spills from water.

## Figures and Tables

**Table 1:** Oil uptake capacities of different adsorbents.

The Name of the Material	Specific Surface Area (m^2^/g)	Oil Uptake per Surface Area (g/m^2^)	Structure
CMP-TST	423.3	0.1665	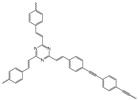
COF-DTF	1056.26	0.164	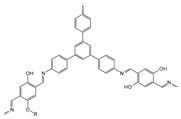
LNU-32	50.6208	0.1456	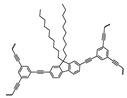
C-600	686	0.1443	–
RGO/PC-2	137.19	0.1262	–
E-POP-1	652.3	0.1257	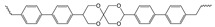
Z8/PC-2	146.84	0.0895	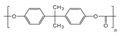
SiO_2_-g-PDAG	70.9	0.0859	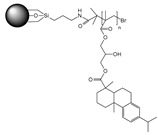
CPOC-302-CF	1782	0.069	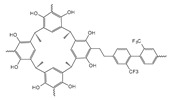
SHMP-1	2100	0.0119	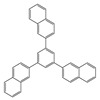
ZIF-8	590	0.0102	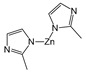
FGO@MOG	1036	0.0048	–

## Data Availability

All data related to this study are presented in this publication.
